# Supplementary material for: Comparative multiomics analysis of cell physiological state after culture in a basket bioreactor
Source: Sci Rep. 2022 Nov 23;12:20161. doi: 10.1038/s41598-022-24687-4 (PMC9686226; doi:10.1038/s41598-022-24687-4)
Supplement: Supplementary file 1 — Supplementary Information 1. [file 41598_2022_24687_MOESM1_ESM.zip › raw data/Transcriptomic raw data/0.SupFile/GOclassification/2.GO_classification_bar.pdf]

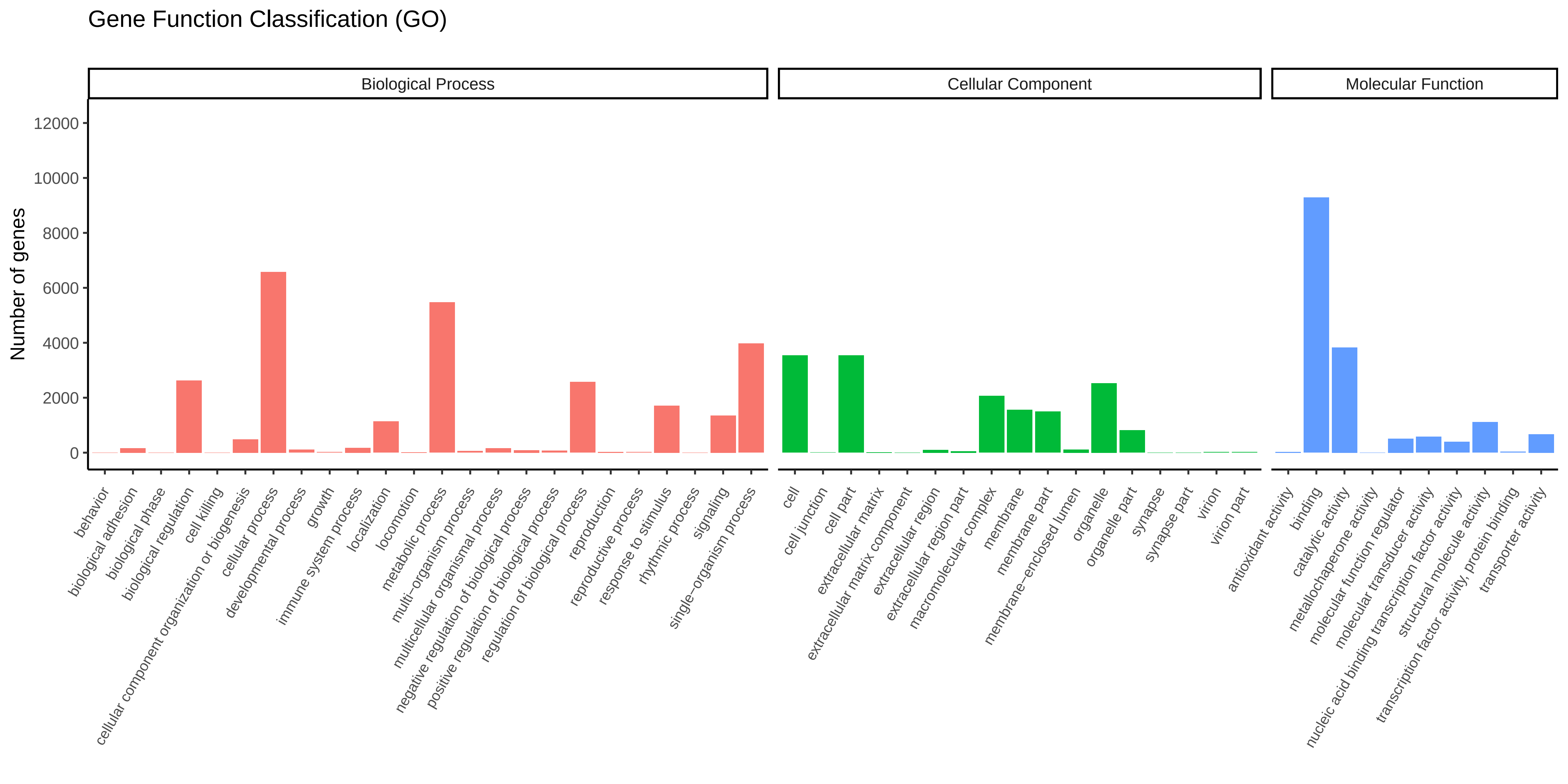

Gene Function Classification (GO)
Biological Process
Cellular Component
Molecular Function
Number of genes
12000
10000
8000
6000
4000
2000
0
behavior
biological adhesion
biological phase
biological regulation
cell killing
cellular component organization or biogenesis
cellular process
developmental process
growth
immune system process
localization
locomotion
metabolic process
multi–organism process
multicellular organismal process
negative regulation of biological process
positive regulation of biological process
regulation of biological process
reproduction
reproductive process
response to stimulus
rhythmic process
signaling
single–organism process
cell
cell junction
cell part
extracellular matrix
extracellular matrix component
extracellular region
extracellular region part
macromolecular complex
membrane
membrane part
membrane–enclosed lumen
organelle
organelle part
synapse
synapse part
virion
virion part
antioxidant activity
binding
catalytic activity
metallochaperone activity
molecular function regulator
molecular transducer activity
nucleic acid binding transcription factor activity
structural molecule activity
transcription factor activity, protein binding
transporter activity
